# Complete Genome Sequence of Highly Adherent *Pseudomonas aeruginosa* Small-Colony Variant SCV20265

**DOI:** 10.1128/genomeA.01232-13

**Published:** 2014-01-23

**Authors:** Denitsa Eckweiler, Boyke Bunk, Cathrin Spröer, Jörg Overmann, Susanne Häussler

**Affiliations:** a Department of Molecular Bacteriology, Helmholtz Centre for Infection Research, Braunschweig, Germany; b Institute for Molecular Bacteriology, TWINCORE GmbH, Centre of Clinical and Experimental Infection Research, Hannover, Germany; c Leibniz Institute DSMZ-German Collection of Microorganisms and Cell Cultures, Braunschweig, Germany

## Abstract

The evolution of small-colony variants within *Pseudomonas aeruginosa* populations chronically infecting the cystic fibrosis lung is one example of the emergence of adapted subpopulations. Here, we present the complete genome sequence of the autoaggregative and hyperpiliated *P. aeruginosa* small-colony variant SCV20265, which was isolated from a cystic ﬁbrosis (CF) patient.

## GENOME ANNOUNCEMENT

The opportunistic pathogen *Pseudomonas aeruginosa* is a key etiological agent of chronic lung infections in cystic fibrosis (CF) patients (1–4). Once CF patients become colonized by *P. aeruginosa*, there is a subsequent gradual deterioration in lung function, which determines the course and prognosis in most CF patients. Only recently have slowly growing subpopulations of bacterial pathogens, termed small-colony variants (SCVs), gained new attention, because an association between their occurrence and persistent infections has been described (5, 6). *P. aeruginosa* SCVs were shown to exhibit increased antibiotic resistance, and their recovery in CF patients was correlated with parameters revealing poor lung function and inhaled antibiotic therapy (7). The *P. aeruginosa* isolate SCV20265 was recovered from the lung of a CF patient from the Hannover Medical School, together with a clonally identical wild-type isolate, which was first described by Häussler et al. in 2003 (8). SCV20265 is hyperpiliated, exhibits an increased twitching motility and capacity for biofilm formation (8, 9), and expresses elevated levels of the bacterial second messenger cyclic diguanylate monophosphate (c-di-GMP) (9, 10). Moreover, the transcriptional and protein profiles of SCV20265 have been recorded (11, 12).

For *de novo* genome assembly, sequence reads were generated by a combination of single-molecule real-time (SMRT) and Illumina sequencing technologies. For the preparation of 10-kb SMRT libraries, ~15 µg unsheared genomic DNA was used. Sequencing was carried out on the PacBio RSII (Pacific Biosciences, Menlo Park, CA) using DNA sequencing reagent 2.0. Illumina libraries were run on a single lane of an Illumina GA IIx with paired 76-base reads, yielding >12.4 million paired-end reads. Genome assembly was performed with the “RS_HGAP_Assembly.1” protocol included in SMRT Portal version 2.0.0, utilizing 219,288 postfiltered reads with an average read length of 4,739 bp. One contig was obtained, which was trimmed, circularized, and adjusted to *dnaA* (PA0001) as the first gene. Quality checks of the final consensus sequence were performed using SMRT View and the Burrows-Wheeler Aligner (BWA) (13), mapping the Illumina reads onto the obtained contig.

The assembled SCV20265 genome consists of a single circular chromosome. At 6,725,183 bp, the size of the SCV20265 genome exceeds the size of 10 out of the 13 *P. aeruginosa* strains for which genomic sequences are available (http://www.pseudomonas.com). The average G+C content is 66.3%, which is consistent with previously sequenced *P. aeruginosa* strains. A total of 6,386 genes, including 12 rRNA and 63 tRNA genes, were annotated with RAST (14). For 3,118 of those genes (48.8%), a clear function (subsystem) could be assigned. Thirteen genomic islands were predicted by IslandViewer analysis (15), six of which were not commonly found in *P. aeruginosa*. While 2 unique genomic islands were related to phage gene transfer, there was an island carrying a type III restriction-modification system and another one containing the Trb conjugation transfer protein cluster. Finally, the *P. aeruginosa* genomic island 7 (PAGI-7) (16) was detected, as well as genes related to arsenic resistance as located on the PACS171b clone fa1382 (17).

### Nucleotide sequence accession number.

The complete SCV20265 genome sequence has been deposited in DDBJ/EMBL/GenBank under the accession no. CP006931.
